# The necessity of healthcare supply chain resilience for crisis preparedness

**DOI:** 10.1177/08404704231207386

**Published:** 2023-11-03

**Authors:** Alexandra M. Wright, Anne Snowdon, Michael Saunders, Dana Trampas

**Affiliations:** 1 8637 University of Windsor, Windsor, Ontario, Canada.; 2 19691 HIMSS, Chicago, Illinois, United States of America.

## Abstract

Prior to and during the COVID-19 pandemic, Canadian provincial health systems and governments did not sufficiently consider healthcare supply chain in their crisis preparedness plans, leading to an exposed and vulnerable healthcare system. There have been many opportunities to learn from past Canadian and global crises, which have emphasized the importance of healthcare supply chain resilience in providing essential care to patients; however, considerations of healthcare supply chain resilience remain a significant gap in preparedness planning. Illustrated through the Canadian response to COVID-19 pandemic, this article will explore how healthcare supply chain resilience should be a necessary consideration in any crisis preparedness plans. Further, without this consideration of healthcare supply chain resilience, it is the person (the patient and healthcare worker), and especially vulnerable populations, that are most put at risk in the event of a future crisis.

## Introduction

Canada continues to experience unexpected disruptions and product shortages for which it is not prepared, leaving the healthcare supply chain system, and therefore the capacity of the healthcare system vulnerable.^24^ From the devastating impact of the COVID-19 pandemic, to continued natural disasters such as wildfires and extreme floods, Canada has had many opportunities to learn, and be prepared for, the next impending crisis, but many provinces and territories have not yet advanced crisis preparedness plans to effectively mitigate risks and respond to crises.^24^ Lack of preparedness creates risks of negative outcomes for Canadians who require access to healthcare services. Ongoing supply disruptions highlight an essential feature of the healthcare supply chain, whereby the end of the healthcare supply chain is human life (e.g., the patient or healthcare worker). Accordingly, destabilization of the healthcare supply chain, particularly during a crisis event, can have catastrophic consequences for human life. In order to mitigate the risk of such consequences, supply chain resilience strategies must be integrated into crisis preparedness planning. During the COVID-19 pandemic, crisis preparedness plans were either not in place or were inadequate, and healthcare supply chain capacity was not sufficient to overcome disruption of critical products, to enable safe delivery of quality healthcare for both patients and for the health workforce.^24-26^ The purpose of this article is two-fold. First, it aims to examine the definition of preparedness and the role healthcare supply chain has played in crisis responses. Secondly, it will present how healthcare supply chain can be understood through the lens of crisis preparedness and crisis preparedness can be strengthened through the integration of supply chain resilience into preparedness efforts.

## Crisis preparedness

Crisis preparedness is defined as “the measures taken by individuals, committees, or organizations to prepare for, prevent, or reduce the impact of a crisis.”^1^ In the vast literature on preparedness planning, this planning is referred to using a variety of terms, such as “emergency planning,”^2,3^ “disaster capacity building,”^4^ and “resilience planning.”^5^ All these terms and concepts focus on preparedness for an unknown crisis.^6^ The “preparedness concept,” Staupe-Delgado and Kruke suggest, typically includes three “minimal attributes”: that preparedness is “active,” “continuous,” and “anticipatory.”^6^ Preparedness, then, is a set of continuous, dynamic activities or actions taken in anticipation of a crisis, so that when a crisis occurs, a system can adequately respond, an important component to the development of resilient systems.^7-9^

## Crisis preparedness plan frameworks

Crisis preparedness is essential for disaster risk reduction and is the focus many popular frameworks to assist governments in developing crisis plans to support the development of resilient systems.^7,8^ There are many common crisis preparedness frameworks, designed to help local and national governments become more prepared for a crisis.^10^ Most notability, crisis preparedness has been identified as a globally important strategy for reducing disaster risk when 197 countries signed on to the Sendai Framework for Disaster Risk Reduction to help countries better prepare for crises.^11^ Another leading framework, developed by World Health Organization (WHO), is the Health Emergency and Disaster Risk Management (H-EDRM) Framework, developed in 2019.^12^ There are also a variety of additional frameworks that are targeted to specific types of crisis incidents, such as influenza or infectious disease.^13,14^

All of the published frameworks have a number of common features of a crisis preparedness strategy, namely, the importance of governance and policy, multi-jurisdictional collaboration, workforce considerations, early warning signals, and prioritizing critical infrastructure such as roads and hospitals. In alignment with these popular frameworks, Staupe-Delgado and Kruke suggest that crisis preparedness typically includes three “minimal attributes,” that should be advanced when formulating crisis preparedness plans: that preparedness is “active,” “continuous,” and “anticipatory.”^6^ In this article, these attributes will be analyzed relative to the role of healthcare supply chain resilience within the context of crisis preparedness.

## Crisis preparedness and health supply chain

Considerations of supply chain do arise in the crisis preparedness literature, but often in the context of emergency management and the “humanitarian supply chain,” or the delivering of urgent supplies to affected areas (such as food, water, and medical supplies).^15^ While these are important in managing a crisis, there is limited, if any, attention to the impact of a crisis on the healthcare system and its capacity to deliver healthcare services not only to those impacted by the emergency but also to the population at large. Below is an illustration of the importance of supply chain to both crisis response, and to support the healthcare system.

Severe Acute Respiratory Syndrome (SARS) was a significant public health crisis in Canada, which illustrated the importance of a stable supply of protective equipment for healthcare workers to safely deliver care. Healthcare workers accounted for 44% of SARS cases in Ontario.^16^ Results of the Justice Campbell Enquiry highlighted that there was “no longer any excuse for health workers not to have available the maximum level of protection through appropriate equipment and training.”^17^ While the recommended stockpiling of critical supplies (e.g., N95 masks and PPE) was implemented by federal agencies and in Ontario, these critical supplies were not adequately rotated into health organizations to mitigate the risk of product expiry, leading to the destruction of 2 million N95 masks in May 2019 by the federal government^18^ and 55 million N95 masks in 2017 by the Ontario Provincial Government.^19^

A similar crisis, Hurricane Maria, shut down several factories in Puerto Rico, where a single manufacturer of Intravenous (IV) bags for North American health systems, Baxter International, was located. This hurricane event led to a severe shortage in intravenous bags required for medication administration across health systems in Canada and the United States. The shortage of the IV bags limited the delivery of critical healthcare services at a time when the United States was going through flu season, increasing the demand for the bags as patients in hospitals required medications to manage their care.^21^ Hospitals relied on this single supplier located in one location (Puerto Rico), resulting in significant risk due to the consequences of a shortage of IV bags that negatively impacted health system capacity to deliver patient care.^21^

In 2005, Hurricane Katrina struck the Gulf Coast and there were significant challenges to the United States Federal Emergency Management Agency’s (FEMA) ability to respond to the crisis and provide the resources and equipment needed, due to an inability to respond quickly enough to get critical supplies to people in the greatest need.^22,23^ Walmart and other “big box stores” stepped in, responding with speed and effectiveness to bring supplies, including food and water, to the affected areas. Walmart was able to provide supplies to hard-hit areas days before FEMA, due to their already resilient supply chain infrastructure and their crisis protocols for large-scale disasters.^22^ Walmart’s experience in supply chain logistics, data and digital infrastructure, and collection of data and information before the storm allowed them to pivot supply chain logistics and transportation before the hurricane unfolded to ensure supplies could be mobilized quickly.^22,23^

## COVID-19 and healthcare supply chain disruption

The most recent crisis that illustrates the critical role of supply chain in crisis preparedness planning was the COVID-19 pandemic. The following summary captures the highlights of a national COVID-19 program of research that examined supply chain capacity in seven provinces (Quebec, Ontario, Newfoundland, Nova Scotia, Alberta, British Columbia, and Manitoba) to document the role of supply chain on health system capacity to respond to the surge in demand for care experienced by Canadians.

Case study findings revealed that all seven provinces studied reacted somewhat slowly to the pandemic, which presented extraordinary challenges with shortages of critical supplies to protect the workforce and to meet the demand for care for patients.^24^ Throughout the pandemic, there was clear evidence of a lack of preparedness among Canadian provinces, particularly preparedness for managing severe shortages of critical products (e.g., PPE).^24-28^

Most, if not all, of the provinces had little or no healthcare supply chain representation at leadership tables or on emergency response teams to provide supply chain expertise and insight into decisions on responses to the pandemic events.^24-29^ Leadership teams were challenged by the lack of data or digital infrastructure necessary to quantify demand utilization rates of critical products and supply inventory volumes to meet demand.^24^ The lack of capacity to understand the rates at which critical supplies were needed, where they were needed most, and where supplies were located, made it nearly impossible to ensure critical products were available when and where they were most needed to safely deliver care to patients infected with COVID-19. Provincial leaders had little choice but to manage the shortage of critical products, especially PPE, by creating allocation frameworks to limit the distribution of PPE to each organization to conserve product use in clinical settings. Without the data and digital infrastructure necessary to inform supply management decisions, PPE was often allocated to areas of assumed need, such as hospitals, leaving Canadians in community settings at the greatest risk (e.g., seniors in Long-Term Care (LTC)) vulnerable and exposed to the virus.^24,29^

The lack of healthcare supply chain capacity and preparedness led to devastating outcomes in Canada. Canada had the highest mortality rates among seniors living in LTC compared to all other countries. Deaths among LTC residents accounted for three percent of all COVID-19 cases in Canada, but 43% of deaths due to COVID-19.^30^ It was recorded that over 2,200 healthcare workers working in LTC contracted COVID-19 between March 2020 and August 2021.^30^ In a 2020 survey of those working in healthcare settings, 51% reported challenges in accessing masks (N95s) and 25% stated that PPE, such as facemasks and goggles, were not available when needed.^31^ Products procured to manage the demand for PPE in health organizations did not always meet Canadian product standards, resulting in significant waste of products that did not meet safety standards. Just one example was the discarding of over 31.1 million masks provided by the Quebec government to day care settings as the masks did not meet minimum safety standards.^32^

SARS, Hurricane Katrina, Hurricane Maria, and the COVID-19 pandemic collectively illustrate the significant role of healthcare supply chain relative to the impact of supply shortages on human life. This evidence suggests that proactive management of supply chain capacity is fundamental to effective crisis preparedness planning. Key features of preparedness planning must consider concepts relevant to supply resilience for health systems in Canada and globally to ensure that every person living in Canada has access to the critical supplies needed to be safe and that health systems have access to critical supplies necessary to have the capacity to deliver care. These critical features of supply chain preparedness are described in the following section.

## Healthcare supply chain resilience and crisis preparedness

Snowdon et al. have defined healthcare supply chain resilience as “the capacity of a healthcare supply chain to support, without interruption or decrease in service level during a crisis, the healthcare system in the delivery of safe and effective healthcare services to populations health systems are mandated to serve.”^33^ Drawing on these findings, in the following section we propose the features of healthcare supply chain resilience that can and should both be understood through the conceptual lens of crisis preparedness and be integrated into crisis preparedness planning, facilitating the measures necessary to supply health system capacity to respond to a crisis appropriately and proactively. We suggest that these features meet the minimal conceptual attributes for preparedness identified by Staupe-Delgado and Kruke,^6^ naturally developing processes that are active, continuous, and anticipatory.

## Healthcare supply chain resilience as active, continuous, and anticipatory

The features of healthcare supply chain resilience are necessarily *active, continuous*, and *anticipatory*, complimenting the need for these attributes in crisis preparedness plans. When approached through the lens of crisis preparedness, we can see how adopting resilient healthcare supply chain practices is necessarily active, continuous, and anticipatory. For example, the creation and maintenance of supply stockpiles enables an active and continuous circulation of critical supplies to offer needed products in anticipation of a crisis. Static or stagnant stockpiles risk having products expire which results in supply becoming unusable during a crisis. Handfield et al. have noted that current approaches to supply management are “dominated by just-in-time efficiencies rather than just-in-case management,” whereby supply of products is sourced to meet current demand with little to no capacity to rapidly respond to surge in demand, which typically happens during a crisis. Canada is especially vulnerable, with a population that is less than .5% of the global population,^36^ and its purchasing power is low compared to the much larger markets of other global countries. In times of crisis, this means that it is unable to compete with the larger global markets, making it incredibly difficult to get necessary supplies in times of disruption.

A move towards healthcare supply chain resilience for crisis preparedness requires adoption of just-in-*case* supply management, and a shift away from “just-in*-time*” approaches focused on lowest cost. Just-in-case management, by its nature, is anticipatory and proactive, as it procures supplies in anticipation of a crisis, disruption, or increase in demand, creating the capacity for proactive and agile response to rapid changes in demand for products to support care delivery. In so doing, it builds supply redundancy through a continuous maintenance of excess supplies and the active circulation and replenishment of this excess stock to enable “just-in-case” response to crises. This anticipatory approach to maintaining “just-in-case” supply inventories offers health systems the capacity to prevent harm to the workforce or patients due to product shortages or disruptions that occur during crises.

A feature critical to supply chain resilience is supply sourcing diversification, or the identification and engagement of multiple suppliers for critical products, particularly domestic suppliers. This approach is a unique pivot from the current one, where most of the healthcare supplies are procured overseas. Diversification should be considered in crisis preparedness planning as supply diversification practices ensures active, continuous, and anticipatory supply chain capacity. Currently, supply disruptions are frequent, and unpredictable, often impacting a variety of product categories. Supply resilience necessarily requires supply chain sources to be actively and continuously diversified in anticipation of a supply disruption. In the event of a crisis or disruption, redundancy in the healthcare supply chain is achieved by diversification of product sources that reduces the risk of supply disruption and enables healthcare systems to continue to deliver care, uninterrupted. Supply chain redundancy and diversification of supply sources are both features of supply chain resilience that can inform crisis preparedness practices and ensure that supplies are always available to support safe patient care delivery.

Maturity of data and digital infrastructure in health systems is crucial for active monitoring of supply chain capacity and proactive decisions to manage risks of supply disruption. Data and digital infrastructure enable transparency of supply chain capacity, whereby flow of data across health systems enables real time tracking of demand for products, utilization rates of product inventory, and supply availability. Digitally enabled supply chain in health systems proactively monitors rates of demand for care and risk of product shortages, particularly for the most vulnerable persons living in Canada. Importantly, data and digital infrastructure inform decisions on the distribution of products based on risk to patients, to ensure products are allocated to those who are most in need, or who are at greatest risk. Data infrastructure enables proactive tracking of supply inventory and demand utilization rates to inform proactive decisions to procure products in anticipation of a surge in demand for care or to manage a supply disruption. Healthcare supply chain data and digital infrastructure therefore meets the minimal conceptual attributes for preparedness including active and continuous monitoring of supply availability and demand, as well as anticipatory decisions to manage future supply needs to prevent disruptions to ensure capacity to deliver care is maintained at all times.

The horizon of healthcare supply chain resilience, when viewed from the conceptual lens of crisis preparedness is not limited to the management of supply disruptions, but rather the elimination of risks due to supply disruptions which are expected to continue well into the future. This is especially important for considerations of healthcare supply chain as any disruption to supply sources results in negative consequences for those in need of care. Supply chain resilience within the context of crisis preparedness enables health system capacity to effectively manage disruptions in supply or sudden increases in demand for care which is common during crisis events. In many respects, then, healthcare supply chain resilience is foundational to crisis preparedness; and, in this way, a crisis preparedness approach to healthcare supply chain resilience both illuminates *how* resilience should be conceived as to be adequately resilient (it must foster practices that are active, continuous, and anticipatory) and why healthcare supply chain resilience should be incorporated into crisis preparedness planning.

## Conclusion

Healthcare supply chain resilience is a critical feature that has been largely missing or limited, in crisis preparedness planning frameworks to date. Health systems assume a critical role in crisis response, as most crises place Canadians at risk due to the heightened demand for health services. By their very nature, crises typically result in destabilized supply of critical products globally. In order to mitigate risks of harm, healthcare supply chains must be sufficiently resilient to ensure that crisis preparedness is proactive and agile, with the capacity to meet the rapid and very dynamic shifts in demands for health services that so often impact Canadians experiencing crisis events. When supply chain considerations are excluded from crisis preparedness planning, then health system preparedness and capacity to respond to disruptions in supply is compromised. Without the supply inventory necessary to deliver care, a health system cannot adequately or equitably deliver safe and quality health services to those who need it most.

This article has described the many examples of devastating crises events which can inform proactive and resilient crises management strategies which include the features of healthcare supply chain resilience to inform crisis preparedness. When healthcare supply chain management is fully incorporated into crisis preparedness planning, then the critical and essential elements of health system capacity to manage crisis is strengthened and health systems can effectively respond to the health needs of all persons living in Canada. The COVID-19 pandemic exposed the vulnerability of Canada's health supply chain capacity to respond to crises, due in large part to the prioritization of “lowest cost” product sourcing and procurement, which resulted in shifting product sources offshore to low labour cost jurisdictions. This “penny-wise, pound foolish” strategy further challenged Canadian leaders with limited market influence (e.g., bargaining power) to source products in a highly competitive global marketplace. For example, where Canada accounts for <2% of the global market for pharmaceutical products.^37^ Despite the impressive efforts of domestic manufacturers to produce critical products in Canada during COVID-19, there remain considerable challenges for these companies in sustaining capacity to produce products if the Canadian market continues to prioritize lowest cost rather than investing in supply diversity which may also contribute to economic growth. Canadian supply chain teams and health system leaders must carefully consider investments in supply chain resilience solutions that meaningfully engage domestic manufacturing production and innovation to enable “just in case” supply management while at the same time achieving diversity in sources of critical products that offers redundancy in the supply chain and strengthens agility in supply chain capacity to adequately respond to crises and to manage the many global supply disruptions Canada continues to experience. Sustainable solutions to the broader supply chain capacity have now become a mission-critical bottleneck that must be overcome, even for the most rudimentary health products such as vaccines, masks, drugs, and devices which are frequently disrupted in global supply chains. To fuel resilience in our health supply chain, Canada must engage multiple and diverse sourcing strategies that achieve supply redundancy that is necessary to mitigate the risks of shortages that compromised the health of so many Canadians. Canadian public policy leaders must overcome short-term views of achieving lowest cost, in favour of investing resources in long-term solutions to address the challenges of supply chain resilience and crisis management on behalf of the Canadian population that demands and expects better preparedness to crises.
